# Adherence to clinical guidelines for the evaluation and management of eosinophilic esophagitis among gastroenterologists in the Arab countries

**DOI:** 10.3389/fped.2025.1521266

**Published:** 2025-04-10

**Authors:** Abdulrahman Al-Hussaini, Jaber Alrashidi, Mohamad Miqdady, Rana Bitar, Isamme AlFayyad

**Affiliations:** ^1^ Division of Pediatric Gastroenterology, Children’s Specialized Hospital, King Fahad Medical City, Riyadh, Saudi Arabia; ^2^ College of Medicine, Alfaisal University, Riyadh, Saudi Arabia; ^3^ Department of Pediatrics, Faculty of Medicine, Prince Abdullah Bin Khalid Celiac Disease Research Chair, King Saud University, Riyadh, Saudi Arabia; ^4^ Department of Pediatrics, Maternity and Children’s Hospital, AlAhsa, Saudi Arabia; ^5^ Division of Pediatric Gastroenterology, Sheikh Khalifa Medical City, Abu Dhabi, United Arab Emirates; ^6^ Research Center, King Fahad Medical City, Riyadh, Saudi Arabia

**Keywords:** eosinophilic esophagitis, survey, clinical guidelines, Saudi Arabia, compliance

## Abstract

**Background:**

The practice patterns of eosinophilic esophagitis (EoE) remain poorly characterized. Few studies investigated the variability of clinical patterns among gastroenterologists, mainly in the United States.

**Objectives:**

We assessed the practice patterns of gastroenterologists in the Arab countries regarding the diagnosis and management of EoE, and their concordance with the European 2017 guidelines and the Proceedings of the AGREE Conference published in 2018.

**Methods:**

We conducted a cross-sectional, self-administered, online survey of practicing gastroenterologists in the Arab countries (April to December 2022). The survey consisted of 23 questions and was designed to assess the respondents’ practice characteristics, knowledge and practice on diagnosis and treatment of EoE.

**Results:**

A total of 190 participants responded to the survey (118 pediatric gastroenterologists and 72 adult gastroenterologists). Thirty-six percent and 31% saw ≥6 new patients with EoE annually, 55% were ‘very familiar’ with the most recent EoE guidelines, and 49% attended ≥3 E-E-related educational activities during the 3 years prior to the survey. The majority of the respondents (72%) did not require a trial of a proton-pump inhibitor (PPI) prior to making the diagnosis of EoE and 66% obtain biopsies from multiple esophageal levels. While 90% of the respondents considered eosinophil-predominant inflammation on esophageal biopsies necessary for the diagnosis of EoE, only 27% felt that symptoms of esophageal dysfunction are necessary for the diagnosis, and only half of the participants considered exclusion of other etiologies of esophageal eosinophilia necessary for the diagnosis of EoE. For first-line treatment, only 16% used PPI monotherapy, 12.6% topical steroids, and 63.5% treat with a variable combination of PPIs, topical steroids, and dietary elimination. Sixty percent would repeat upper endoscopy to determine histologic improvement and 72% use maintenance therapy in responders. Compared to pediatric gastroenterologists, significantly fewer adult gastroenterologists reported taking biopsies from proximal and distal esophagus (34% vs. 66%) and gastric and duodenal biopsies (67% vs. 90%) when EoE was suspected (*P* < 0.001).

**Conclusion:**

There is significant variability in adherence to EoE guidelines among gastroenterologists in the Arab countries. Our results highlight areas of need for continuous education and form the basis to assess implementation efforts in the future.

## Introduction

Eosinophilic esophagitis (EoE) is a chronic immune/antigen-mediated esophageal inflammatory disease associated with esophageal dysfunction resulting from eosinophil-predominant inflammation [≥15 eosinophils per high power field (eos/hpf)] (1, 2). There has been remarkable progress in the understanding of EoE basic immune mechanisms, natural history, and clinical phenotypes since the time of its first recognition more than 3 decades ago. The diagnostic and treatment approaches have evolved over time. As a result, criteria for diagnosis established in the 2007 consensus recommendations (2) were further refined in the 2011 (3), 2013 (4), 2014 (5), 2017 (6), 2018 (7), and 2024 (8) updates to the consensus recommendations. The use of PPI was revised in the European EoE guidelines (6) and the proceedings of the AGREE conference (7), from a requirement to make a diagnosis of EoE to be the first-line treatment.

In the setting of these evolving guidelines, real-world practice patterns concerning the diagnosis and management of EoE remain poorly characterized. Few studies reported on the variability of clinical patterns among pediatric and adult gastroenterologists in the management and diagnosis of EoE and the adherence to the published guidelines. Most of these studies investigated the practice patterns among gastroenterologists in United States (9–15).

In the Arab world, there is limited data on EoE (16–26). Although these reports described several aspects of EoE disease in Saudi Arabia, none investigated the patterns of clinical practice and adherence to guidelines. The understanding of practice patterns is essential to prioritize training efforts, identify continuing education needs, assess implementation efforts, and improve quality of care regarding EoE diagnosis and management in the Arab countries. The objectives of our study were: (1) to assess how pediatric and adult gastroenterologists across the Arab countries diagnose, evaluate, and treat patients with EoE and how they adhere to the clinical guidelines recommended by higher world authorities and societies on diagnosis and management of EoE; (2) to identify gaps in knowledge and the continuing education needs of local gastroenterologists.

## Patients and methods

### Study design and setting

This was a cross-sectional study of pediatric and adult gastroenterologists in the Arab countries using a self-administered online survey, created using “Google forms”. The survey was conducted between April and December 2022.

### Recruitment of the study participants

A link to a web-based survey was distributed via professional electronic network to pediatric and adult gastroenterologists practicing in the Arab countries. There are two professional electronic networks: one belongs to the members of the Saudi society of pediatric gastroenterology, hepatology and nutrition (SASPGHAN) and included 130 pediatric gastroenterologists, the other group belongs to the members of the Saudi society of gastroenterology (SGA) and included 350 adult gastroenterologists. Although the majority of the members in the 2 groups are Saudis, but both groups included gastroenterologists practicing in the Arab countries. We also used e-mail to reach gastroenterologists in the Arab countries not included in the two professional electronic networks.

### Survey design

The survey was designed to assess EoE practice patterns of gastroenterologists and adherence to the consensus clinical guidelines for both children and adults endorsed by the European EoE guidelines (6) and the proceedings of the AGREE conference (7). The survey comprised of 23 questions over 4 main categories: (1) respondent and practice characteristics, (2) EoE symptoms and endoscopic features; (3) EoE diagnosis, and (4) EoE treatment (Supplementary Table 1). Before distribution, the survey questions were sent to 5 pediatric and adult gastroenterologists to assess for language clarity, comprehensibility and appropriateness of the survey content. In addition, we conducted a pilot study with 20 participants who completed the survey, in order to assess the clarity of the survey questions, the feasibility of data collection, and the estimated time required for completion. Based on the feedback from the pilot test, minor modifications were made to improve question clarity. The final version of the survey was then used for data collection in the main study.

### Statistical analysis

Data were analyzed using SPSS version.25 (IBM Corp, Armonk, NY, USA). Descriptive statistics [Frequencies/proportions, means ± standard deviation (SD)] were used to summarize the characteristics of participating physicians, diagnosis and treatment of EoE. Chi-square/Fisher's exact tests were used (where applicable) to compare EoE with the physicians’ specialty, practice setting, number of EoE activities attended, and duration of practice. A statistical level of significance set at *p* < 0.05.

### Ethical approval

IRB approval was obtained prior to study conduct. No individual participant identifiers were collected. All responses were anonymous.

## Results

### Respondents and practice characteristics

A total of 190 gastroenterologists from different Arab countries completed the survey, 122 (64.2%) of whom were from Saudi Arabia and 40 (21%) were from United Arab Emirates; the remaining 28 participants were from 11 different Arab countries. Of the pediatric gastroenterologists who were members of the SASPGHAN, 82 of 130 (63%) completed the survey, and of the adult gastroenterologists who were members of the SGA, 40 of 350 completed the survey (11.4% response rate). The background characteristics, practice of survey respondents, years of clinical experience, volume of patients with EoE managed annually, familiarity with EoE consensus guidelines, and number of EoE-related educational activities attended are shown in Table 1.

**Table 1:** Overall characteristics of the respondents.

Variables	Respondents = 190
1. Practice setting	
1. Governmental, tertiary care (University-based)	56 (29.5%)
2. Governmental, tertiary care (Non-University based)	72 (38%)
3. Governmental, secondary care	29 (15%)
4. Private practice	33 (17.5%)
2. How many years in practice?	
1. 0–5 years	65 (34%)
2. 6–10 years	33 (17.5%)
3. 11–20 years	48 (25%)
4. >20 years	44 (23%)
3. Region of practice	
Saudi Arabia	122 (65%)
United Arab Emirates	40 (21%)
Jordan	4 (2%)
Syria	4 (2%)
Algeria	2 (1%)
Tunisia	1 (0.5%)
Egypt	1 (0.5)
Lebanon	2 (1%)
Morocco	2 (1%)
Oman	5 (2.5%)
Kuwait	5 (2.5%)
Sudan	1 (0.5%)
Iraq	1 (0.5%)
4. Your EoE practice population	
Adults only	55 (29%)
Children only (age ≤14 years)	118 (62%)
Mixture of adults and children	17 (9%)
5. Are you familiar’ with EoE consensus guidelines?	
Very familiar	104 (54.7%)
Somewhat familiar	81 (42.6%)
Not familiar	5 (2.7%)
6. Number of EoE-related educational activities attended in the previous 3 years	
None	23 (12%)
1–2	73 (38.3%)
3–4	51 (26.7%)
≥ 5	43 (22%)
7. Areas of sub-specialization	
General gastroenterology	91 (48%)
Hepatology/transplant hepatology	37 (19.5%)
Advanced endoscopy	41 (21.5%)
Inflammatory bowel disease	55 (29%)
Motility disorders	18 (9.5%)
Nutrition	23 (12%)
8. New EoE patients do you diagnose annually	
None	5 (2.6%)
1–5	116 (61%)
6–15	56 (29.5%)
16–25	10 (5.4%)
>25	3 (1.5%)

### Knowledge of EoE symptoms and endoscopic features

Dysphagia (95% of respondents) and food impaction (87%) were the most common symptoms considered when diagnosing EoE. Other common presenting findings considered included individual or family history of atopic disorders/food allergies (67%), refractory reflux (50%), and chest pain and vomiting (46%). In terms of endoscopic findings, linear furrows (94%) and esophageal rings (88%) were the most common endoscopic features that respondents associated with the diagnosis of EoE, followed by white plaques/exudates (81.5%), esophageal stricture (77.4%), and narrow caliber esophagus (62.5%) (Table 2).

**Table 2:** Diagnosis of eosinophilic esophagitis (questions 9–17).

Variables	Respondents = 190		
9. Symptoms would make you consider the diagnosis of EoE			
(a) Heartburn	59 (31%)		
(b) Regurgitation	43 (22.5%)		
(c) Refractory reflux	94 (49.5%)		
(d) Chest pain	88 (46%)		
(e) Abdominal pain	36 (19%)		
(f) Dysphagia	181 (95%)		
(g) Odynophagia	69 (36.4%)		
(h) Food impaction	166 (87.3%)		
(i) Nausea	27 (14%)		
(j) Vomiting	88 (46%)		
(k) Weight loss/failure to thrive	80 (42%)		
(l) Anemia	26 (13.7%)		
(m) Hematemesis	28 (14.7%)		
(n) Personal or family history of atopic disorders/food allergies	127 (67%)		
10. Endoscopic findings do you consider consistent with the diagnosis of EoE			
(a) Esophageal rings	167 (88%)		
(b) Esophageal stricture	147 (77.4%)		
(c) Esophageal ulcer	38 (20%)		
(d) Esophageal nodule	32 (17%)		
(e) Esophageal mass	9 (4.7%)		
(f) Narrow caliber esophagus	119 (62.5%)		
(g) Linear furrows	178 (94%)		
(h) White plaques/exudates	155 (81.5%)		
(i) Erosive esophagitis	51 (27%)		
(j) Decreased mucosal vascularity	61 (32%)		
(k) Congested esophageal mucosa	58 (30.5%)		
(l) Mucosal tears after passing the endoscope	76 (40%)		
(m) Hiatal hernia	8 (4%)		
(n) Normal appearing esophagus	52 (27%)		
11. Do you require that a patient is on PPI diagnosis of EoE?			
1. Yes	58 (20.5%)		
2. No	137 (72%)		
12. Number of esophageal biopsies to diagnose of EoE			
1. 2	7 (3.6%)		
2. 4	63 (33%)		
3. 6	92 (48.4%)		
4. 8	24 (12.6%)		
5. >8	9 (4.7%)		
13. From where in the esophagus do you take biopsies?			
1. Proximal esophagus	136 (71.5%)		
2. Mid esophagus	158 (83%)		
3. Distal esophagus	175 (92%)		
14. Do you put biopsies in different pathology jars?			
(a) Yes	159 (83.5%)		
(b) No	36 (19%)		
15. Do you get biopsies from stomach and duodenum?			
(a) Stomach only	31 (16.3%)		
(b) Duodenum only	5 (2.6%)		
(c) Both stomach and duodenum	159 (83.5%)		
16. Cut off eosinophils/high-power field do you use for diagnosis of EoE			
(a) 10	6 (3%)		
(b) 15	149 (78.4%)		
(c) 20	21 (10.5%)		
(d) 25	10 (5.2%)		
(e) I don’t use a specific cut off point	9 (4.67%		
17. Which of the following are necessary to diagnose EoE?	Not necessary	Helpful, but not necessary	Necessary
1. Clinical symptoms of esophageal dysfunction	10 (5.2%)	129 (68%)	51 (26.8%)
2. Allergy testing	67 (35.3%)	119 (62.7%)	4 (2%)
3. Barium swallow study	80 (42.1%)	105 (55.3%)	5 (2.6%)
4. Eosinophil-predominant inflammation on esophageal biopsy	2 (1%)	17 (9%)	173 (90%)
5. Peripheral eosinophilia	75 (39.5%)	110 (58%)	5 (2.5%)
6. Exclusion of secondary causes of esophageal eosinophilia	21 (11%)	70 (37%)	99 (52%)
7. Ruling out gastroesophageal reflux disease with pH testing	93 (49%)	83 (43.6%)	14 (7.4%)
8. No clinical response to a PPI trial	52 (27.5%)	111 (59.5%)	23 (12%)
9. Personal or family history of atopic disorders/food allergies	13 (6.4%)	173 (91.6%)	4 (2%)

### Knowledge of the guidelines related to EoE diagnosis

Consistent with the EoE guidelines published at the time of our study (6, 7), 72% of the respondents did not require a PPI trial prior to the diagnostic endoscopy. Regarding biopsy procurement, guidelines recommend obtaining at least 6 biopsies from different levels of the esophagus (at least two samples from each level), placing the biopsies in separate jars, and obtaining duodenal and gastric mucosal biopsies at initial diagnosis to exclude eosinophilic gastroenteritis. Most of the respondents adhered to the guidelines: 65.7% obtained at least 6 biopsies from different levels; 83.5% place biopsies from different locations in separate jars and obtained biopsies from the stomach and duodenum. The majority of respondents (78.4%) used a cut-off point of ≥15 eos/hpf on their esophageal mucosal biopsy specimens for histopathologic diagnosis (Table 2).

The recent definition of EoE in the recent guidelines included combination of the following 3 criteria: (1) consistent symptoms of esophageal dysfunction; (2) an esophageal biopsy specimen with at least 15 eos/hpf; and exclusion of other systemic and local causes of esophageal eosinophilia (6, 7). While 90% of the respondents considered eosinophil-predominant inflammation on esophageal biopsies necessary for the diagnosis EoE, only 27% felt that symptoms of esophageal dysfunction are necessary for the diagnosis, and only half of the participants considered exclusion of other etiologies of esophageal eosinophilia necessary for the diagnosis of EoE. More than 90% of the did not consider allergy testing, barium swallow study, peripheral eosinophilia, ruling out GERD by a pH study, or individual and family history of atopic disorders/food allergies necessary for the diagnosis of EoE.

### Knowledge of the guidelines related to EoE treatment

**Figure 1:**
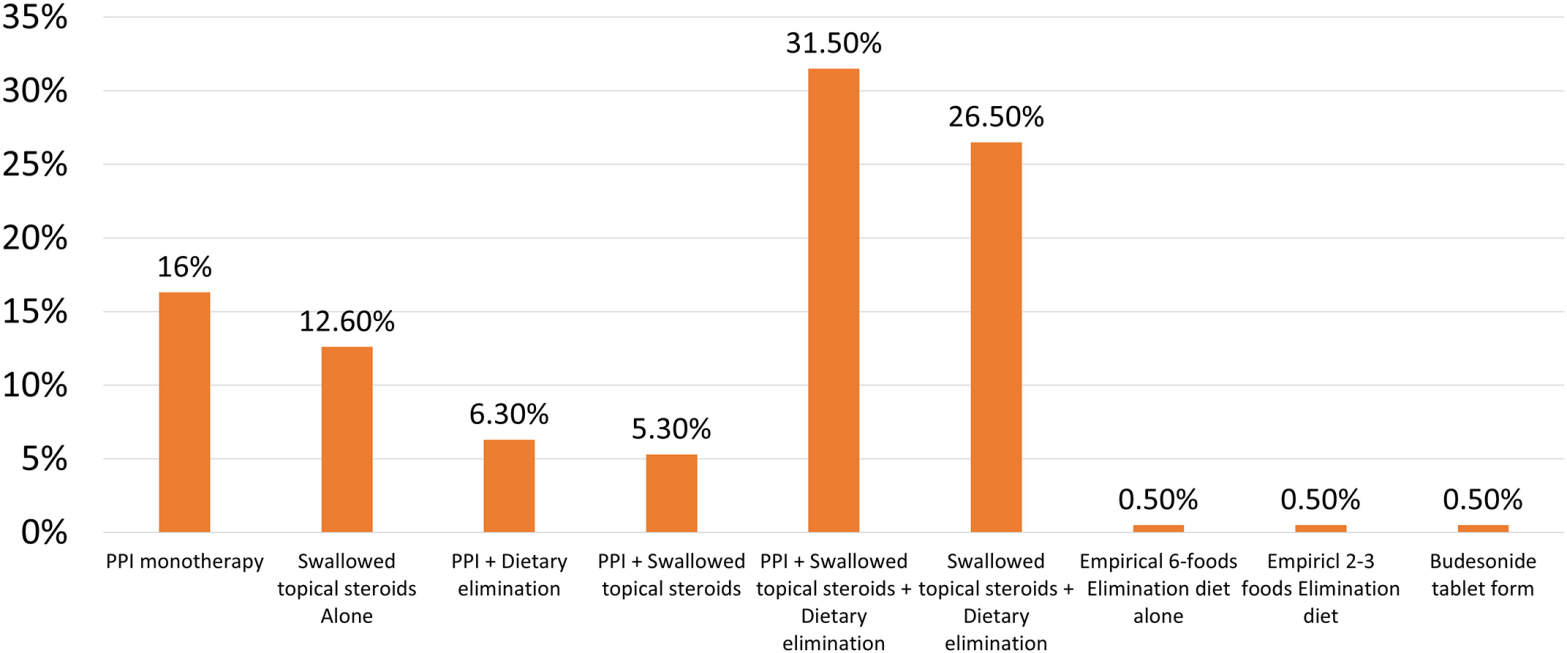
The frequency of prescribing one or more treatment modalities as first-line therapy of EoE by Arab gastroenterologists.

Of the first-line treatments selected by respondents, only 16% recommended using PPI alone as initial therapy; majority used topical steroids, either alone (12.6%) or in various combinations with PPI and dietary elimination (63.5%) (Figure 1). Dietary elimination was rarely selected as a single first-line treatment. Clinical guidelines recommend involving the patient (or parents) in the decision-shared process; 85% of the respondents adhered to this principle. In response to a question, what do you do to determine the success of first line therapy? 60% of the participants would repeat upper endoscopy with biopsies to determine histologic improvement, and 35% would repeat upper endoscopy only if symptoms are persistent; the remaining 5% would repeat upper endoscopy just to evaluate the endoscopic appearance without biopsies.

**Figure 2:**
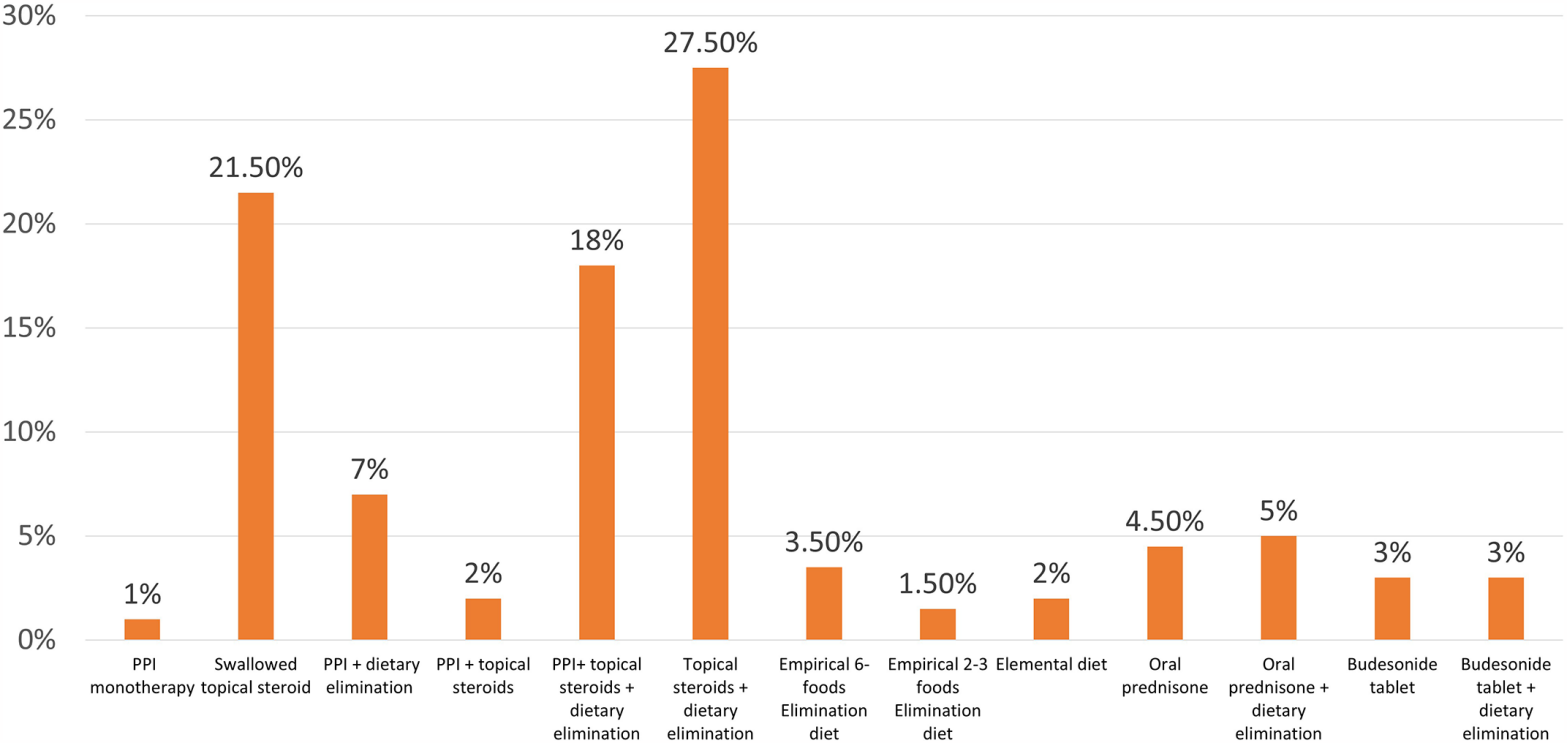
The frequency of prescribing one or more treatment modalities as second-line therapy of EoE by Arab gastroenterologists.

For second-line therapy in case of failure of first-line treatment, there was a wide range of answers (Figure 2). The majority of the respondents (69%) used swallowed topical steroids, either alone (21.5%) or in combination with dietary elimination and PPI; a minority of participants used an elemental diet, systemic steroids, or budesonide tablet. In response to a question: in patients who responded to therapy, do you use maintenance therapy? 72% responded “yes”, 7% answered “no”, and 21% responded “sometimes”. In response to a question describing a patient suspicious of EoE with a narrow caliber esophagus during initial endoscopy, 63% of respondents would wait to confirm the diagnosis of EoE and perform dilation only if the patient was symptomatic after initial medical therapy; 27% would dilate at the first endoscopy if it was severe stricture.

### Comparison of practices among gastroenterologists based on specialty and work setting

Differences in clinical practices among Arab gastroenterologists were explored (Supplementary Tables 1–S4). Stratifying the responders by their specialty revealed that 62% were pediatric gastroenterologists. The main differences were in the practices to obtain biopsies with pediatric gastroenterologists who reported taking both gastric and duodenal biopsies when suspecting EoE more often than adult gastroenterologists (90% vs. 67%, *p* = 0.001), sampling more esophageal levels (66.4% vs. 34.2%, *p* < 0.001), and placing them in different jars (92% vs. 64.4%, *P* < 0.001) (Supplementary Table 1). On the other hand, adult gastroenterologists involved patients in the decision-making process more frequently than pediatric gastroenterologists (81% vs. 92%, *P* = 0.043).

Stratifying the participants by their practice setting revealed that academic gastroenterologists were more adherent to get minimum of 4 esophageal biopsies to diagnose EoE than gastroenterologists working in non-academic institutes (77% vs. 59%, *P* *=* 0.019). No significant differences in other clinical practices were found between the two groups (Supplementary Table 2).

Stratifying the responses by the number of EoE-related educational activities the participants attended in the previous 3 years revealed that the participants who attended ≥ 3 educational activities were more adherent to clinical guidelines that recommend using maintenance therapy (78.4% vs. 65.3%, *P* *=* 0.043) and dilating severe esophageal stricture (35% vs. 19.4%, *P* *=* 0.014) (Supplementary Table 3).

Stratifying the responders by their years in practice revealed that junior gastroenterologists (< 10 years in practice) used a cut-off point of ≥15 eos/hpf for diagnosis of EoE more frequently than senior gastroenterologists (89% vs. 68%, *P* = 0.001). No significant differences in other clinical practices were found between the two groups (Supplementary Table 4).

## Discussion

Our data showed that the real-life practices of gastroenterologists in the Arab countries were adhered to the EoE consensus guidelines (6, 7) in some aspects and deviated from the guidelines in other respects. Specifically, they were non-adherent to the guidelines in two main aspects. First, only 27% of the participants felt that symptoms of esophageal dysfunction are necessary for diagnosis, as compared to 58% of respondents in a survey study among American gastroenterologists (12). In the American cohort, 63% of the respondents considered exclusion of other etiologies of esophageal eosinophilia necessary for diagnosis, as compared to 50% of respondents in our study Although the cut-off value of 15 eos/hpf on esophageal biopsy as a diagnostic criterion remain unchanged, the importance of considering the symptoms of esophageal dysfunction and exclusion of other causes of eosinophilia have been emphasized as diagnostic criteria of EoE in the more recent guidelines (6, 7). Second, only 16% recommended using PPI alone as initial therapy, as compared to 61% of respondents to the survey in United States (13). This finding is corroborated with the low rate of use of PPI as initial therapy in the local EoE studies (17–25). On the other hand, most gastroenterologists in the Arab countries were following the best available evidence in many areas related to the diagnosis and management of EoE. Specifically, the majority (>80%) recognize the presenting symptoms and common endoscopic findings associated with the diagnosis of EoE. Second, 72% of the respondents did not require a PPI trial prior to the diagnostic endoscopy. Third, most Arab gastroenterologists (66% to 84%) adhered to the biopsy guidelines. Fourth, more than 90% of the respondents did not consider allergy testing, barium swallow study, peripheral eosinophilia, ruling out GERD by a Ph study, or individual and family history of atopic disorders/food allergies necessary for the diagnosis of EoE. Furthermore, 85% would involve the patient (or parents) in the decision-shared process. Finally, many gastroenterologists (60%) would repeat endoscopy and obtain biopsies to determine the success of first-line therapy, as compared to 16% (11) and 45% (13) of American gastroenterologists. This practice was reflected in most of the regional studies published on EoE that showed a common practice of confirming response to treatment endoscopically and histopathologically (17–25).

Our study is the first to investigate the real-life practice patterns of gastroenterologists in the evaluation and management of EoE after the publication of the European guidelines in 2017 (5) and the guidelines endorsed by a group of experts in the AGREE conference in 2018 (6). Previous studies have evaluated the practice patterns of gastroenterologists based on the older EoE clinical guidelines (2–5) and have demonstrated that adherence to guidelines was poor (12%) among gastroenterologists in many areas related to the diagnosis and management of EoE (11). On the other hand, our study showed that most of Arab gastroenterologists (>50%) were adherent to the EoE guidelines. There are several reasons that could explain the better adherence of Arab gastroenterologists, which likely related to the characteristics of the cohort of physicians responding to our survey. First, 50% of the respondents attended ≥3 educational activities about EoE during the 3 years prior to the survey. Greater attendance to educational activities correlated to the recommendation of using maintenance therapy after steroid response and dilation of severe esophageal strictures observed during the first endoscopy, which demonstrates the effectiveness and influence of attending the EoE-related workshops and lectures on physician practice. The increased confidence in managing patients with EoE among respondents who attended more workshops and lectures emphasizes the importance of providing continuous education on EoE in national gastroenterologic societal meetings and the need for a wider dissemination of EoE practice guidelines and implementing structured EoE training programs. Second, 55% of the respondents in our study reported being “very familiar” with the EoE consensus guidelines as compared to 24% in a survey study from United States (11); hence, it is possible that many of gastroenterologists in our region with great interest in EoE responded to the survey. Third, most of the participants were pediatric gastroenterologists (62%). Our data and those of other studies (9, 15) showed that pediatric gastroenterologists were more adherent to the EoE consensus guidelines than adult gastroenterologists. All the above-mentioned reasons might have introduced recruitment bias and led to higher adherence rates than in previous studies conducted in the West.

In our study, as well as other previous studies, the most surveyed area in clinical practice that showed marked variability in responses was EoE treatment. Such variability in responses is not surprising because of the lack of well-designed randomized controlled trials that compare different treatment options. As a result, most gastroenterologists would select treatment modality merely based on their experience and patient preference. Because of the rapidly evolving research and better understanding of EoE, the treatment approach has changed over time. The use of PPI was revised from a requirement to make a diagnosis of EoE in 2007, 2011 and 2014 guidelines (2, 3, 5) to be the first-line treatment of EoE in the 2017 and 2018 guidelines (6, 7). These changing guidelines likely caused considerable confusion among gastroenterologists and led to poor adherence to the guidelines. More recently, in August 2024, an update of the recommendations was generated by ESPGHAN and revised the options for initial drug treatment to recommend PPI, diet, or topical steroids might be offered as first line anti-inflammatory therapy (8). Also, the updated guidelines recommend the use of biologics as a part of the potential armamentarium for difficult cases with EoE that do not respond to or intolerant of conventional therapies, and systemic steroids may be considered as the initial treatment for esophageal strictures before esophageal dilation (8). For second line therapy of EoE, the different responses and inconsistent practices of gastroenterologists in our study reflect the various options available to treat EoE and the lack of clinical trials that compare different treatment modalities to guide the decision-making process.

In addition to the recruitment bias mentioned above, other limitations of our study include the low response rate of adult gastroenterologists (11.4%). In addition, the majority of respondents were from Saudi Arabia and United Arab Emirates (86%). These two characteristics may limit the generalizability of our results. Our study was conducted prior to the dissemination of the most recent 2024 ESPGHAN guidelines, therefore we were not able to assess the participants’ practice of use of biologics in the treatment of EoE. Another limitation is that “multivariate analysis was not performed for identifying the predictors of adherence to guidelines”.

In conclusion, our results highlight areas of need for continuous education for gastroenterologists in the Arab countries and form the basis to assess implementation efforts in the future.

## Data Availability

The original contributions presented in the study are included in the article/Supplementary Material, further inquiries can be directed to the corresponding author.

## References

[1] Massironi S Elvevi A Panceri R Mulinacci G Colella G Biondi A Eosinophilic esophagitis: does age matter? Expert Rev Clin Immunol. (2024) 20(2):211–23. 10.1080/1744666X.2023.2274940 37870118

[2] Furuta GT Liacouras CA Collins MH Gupta SK Justinich C Putnam PE Eosinophilic esophagitis in children and adults: a systematic review and consensus recommendations or diagnosis and treatment. Gastroenterology. (2007) 133:1342–63. 10.1053/j.gastro.2007.08.017 17919504

[3] Liacouras CA Furuta GT Hirano I Atkins D Attwood SE Bonis PA Eosinophilic esophagitis: updated consensus recommendations for children and adults. J Allergy Clin Immunol. (2011) 128:3–20. 10.1016/j.jaci.2011.02.040 21477849

[4] Dellon EV Gonsalves N Hirano I Furuta GT Liacouras CA Katzka DA. ACG clinical guideline: evidenced based approach to the diagnosis and management of esophageal eosinophilia and eosinophilic esophagitis (EoE). Am J Gastroenterol. (2013) 108:679–92. 10.1038/ajg.2013.71 23567357

[5] Papadopoulou A Koletzko S Heuschkel R Dias JA Allen KJ Murch SH Management guidelines of eosinophilic esophagitis in childhood. J Pediatr Gastroenterol Nutr. (2014) 58:107–18. 10.1097/MPG.0b013e3182a80be1 24378521

[6] Lucendo AJ Molina-Infante J Arias Á von Arnim U Bredenoord AJ Bussmann C Guidelines on eosinophilic esophagitis: evidence-based statements and recommendations for diagnosis and management in children and adults. United European Gastroenterol J. (2017) 5(3):335–58. 10.1177/2050640616689525 28507746 PMC5415218

[7] Dellon EV Liacouras CA Molina-Infante J Furuta GT Spergel JM Zevit N Updated international consensus diagnostic criteria for eosinophilic esophagitis: proceedings of the AGREE conference. Gastroenterology. (2018) 155:1022–33. 10.1053/j.gastro.2018.07.009 30009819 PMC6174113

[8] Amil-Dias J Oliva S Papadopoulou A Thomson M Gutiérrez-Junquera C Kalach N Diagnosis and management of eosinophilic esophagitis in children: an update from the European society for paediatric gastroenterology, hepatology and nutrition (ESPGHAN). J Pediatr Gastroenterol Nutr. (2024) 79:394–437. 10.1002/jpn3.12188 38923067

[9] King J Khan S. Eosinophilic esophagitis: perspectives of adult and pediatric gastroenterologists. Dig Dis Sci. (2010) 55:973–82. 10.1007/s10620-009-0801-9 19390967

[10] Shaffer C Ghaffari G. International survey on evaluation and management of eosinophilic esophagitis. World Allergy Organ J. (2012) 5(9):95–102. 10.1097/WOX.0b013e3182690759 23283208 PMC3694720

[11] Eluri S Iglesia EGA Massaro M Peery AF Shaheen NJ Dellon ES. Practice patterns and adherence to clinical guidelines for diagnosis and management of eosinophilic esophagitis among gastroenterologists. Dis Esophagus. (2020) 33(7):doaa025. 10.1093/dote/doaa025 32378700 PMC7350163

[12] Peery AF Shaheen NJ Dellon ES. Practice patterns for the evaluation and treatment of eosinophilic esophagitis. Aliment Pharmacol Ther. (2010) 32(11-12):1373–82. 10.1111/j.1365-2036.2010.04476.x 21050240 PMC3099135

[13] Chang JW Saini SD Mellinger JL Chen JW Zikmund-Fisher BJ Rubenstein JH. Management of eosinophilic esophagitis is often discordant with guidelines and not patient-centered: results of a survey of gastroenterologists. Dis Esophagus. (2019) 32(6):doy133. 10.1093/dote/doy133 30715230 PMC6561423

[14] Spergel JM Book WM Mays E Song L Shah SS Talley NJ Variation in prevalence, diagnostic criteria, and initial management options for eosinophilic gastrointestinal diseases in the United States. J Pediatr Gastroenterol Nutr. (2011) 52(3):300–6. 10.1097/MPG.0b013e3181eb5a9f 21057327 PMC4450826

[15] Zifman E Banai H Shamir R Ringel-Kulka T Zevit N. Practice differences in the diagnosis and management of eosinophilic esophagitis among adult and pediatric gastroenterologists in Israel. J Pediatr Gastroenterol Nutr. (2018) 67(1):34–9. 10.1097/MPG.0000000000001909 29394215

[16] Al-Hussaini A Smaan T Elhag E. Esophageal trachealization: a feature of eosinophilic esophagitis. Saudi J Gastro. (2009) 15(3):193–5. 10.4103/1319-3767.54747 PMC2841420 19636182

[17] Al-Hussaini A Al-Idressi I Al-Zahrani M. The role of allergy evaluation in children with eosinophilic esophagitis. J of Gastroenterol. (2013) 48:1205–12. 10.1007/s00535-012-0741-6 23354622

[18] Al-Hussaini A Seaman T Hag IE. Eosinophilic esophagitis in a developing country: is it different from developed countries? Gastroenterol Res Pract. (2013):526037. 10.1155/2013/526037 24371436 PMC3858865

[19] Al-Hussaini A AboZeid A Hai A. How does esophagus look on barium esophagram in pediatric eosinophilic esophagitis? Abdom Radiol. (2016) 41(8):1466–73. 10.1007/s00261-016-0712-0 PMC4972850 27010937

[20] Al-Hussaini A. Savary dilation effective and safe treatment for esophageal narrowing associated with pediatric eosinophilic esophagitis. J Pediatr Gastroenterol Nutr. (2016) 63:474–80. 10.1097/MPG.0000000000001247 27111342 PMC5084639

[21] Hasosah MY Sukkar GA Alsahafi AF Thabit AO Fakeeh ME Al-Zahrani DM Eosinophilic esophagitis in Saudi children: symptoms, histology and endoscopy results. Saudi J Gastroenterol. (2011) 17:119–23. 10.4103/1319-3767.77242 21372349 PMC3099057

[22] Saadah OI Aburiziza AJ Abu Shakra RI. Eosinophilic esophagitis in children from western Saudi arabia: relative frequency, clinical, pathological, endoscopic, and immunological study. Gastroenterol Res Pract. (2012):328253. 10.1155/2012/328253 23304124 PMC3529483

[23] Saeed A Assiri AM Al Asmi M Ullah A. Trend, clinical presentations and diagnosis of eosinophilic esophagitis in Saudi children. Saudi Med J. (2018) 39:668–73. 10.15537/smj.2018.7.22425 29968888 PMC6146256

[24] Alzahrani M Alshehri M Rassam L Alrobaie K Al Mubarak D Asiri M Epidemiologic characteristics and clinical pattern of eosinophilic esophagitis: single centre experience. Bahrain Med Bullet. (2022) 44(1):1–5.

[25] Alkhowaiter S. Eosinophilic esophagitis. Saudi Med J. (2023) 44(7):640–6. 10.15537/smj.2023.44.7.20220812 37463709 PMC10370381

[26] Fahad A Musthafa P Mohammed A Nahla A Majid A Evan D Eosinophilic esophagitis: current concepts in diagnosis and management. Saudi J Gastroenterol. (2024) 30:210–27. 10.4103/sjg.sjg_50_24 38752302 PMC11379248

