# Supplementary material for: Adherence to clinical guidelines for the evaluation and management of eosinophilic esophagitis among gastroenterologists in the Arab countries
Source: Front Pediatr. 2025 Apr 10;13:1521266. doi: 10.3389/fped.2025.1521266 (PMC12018459; doi:10.3389/fped.2025.1521266)
Supplement: Supplementary file 1 [file Table1.docx]

**Supplementary table 1.** EoE practice patterns based on specialty

| Guidelines recommendations | Adult GI  n = 72 | Ped GI  n = 118 | P-value |
| --- | --- | --- | --- |
| 1. No need for PPI trial prior to diagnosis of EoE? (%) | 55 (75.3) | 82 (67.2) | 0.229 |
| 1. Number 6 of esophageal biopsies to diagnose of EoE (%) | 43 (58.9) | 82 (67.4) | 0.242 |
| 1. Biopsies from Proximal and distal esophagus (%) | 25 (34.2) | 81 (66.4) | **<0.001** |
| 1. Place biopsies from different locations in different jars (%) | 47 (64.4) | 112 (91.8) | **<0.001** |
| 1. Biopsies from stomach and duodenum on initial exam (%) | 49 (67.1) | 110 (90.2) | **0.001** |
| 1. Use of cut point of ≥15 eosinophils /hpf for diagnosis (%) | 55 (75.3) | 98 (80.3) | 0.412 |
| 1. Necessity for symptoms + positive biopsy + exclusion of secondary causes | 9 (12.3) | 22 (18.0) | 0.292 |
| 1. PPI monotherapy as first line treatment (%) | 49 (67.1) | 73 (59.8) | 0.309 |
| 1. Involvement of patients (or parents) in the decision-shared process (%) | 67 (91.8) | 99 (81.1) | **0.043** |
| 1. Assess both symptoms and histology as markers of treatment response (%) | 42 (57.5) | 74 (60.7) | 0.667 |
| 1. Use of maintenance therapy after steroid response (%) | 51 (69.9) | 89 (73.0) | 0.643 |
| 1. Dilation of severe esophageal strictures seen during first endoscopy (%) | 16 (21.9) | 37 (30.3) | 0.201 |
| 1. **Are you familiar’ with EoE consensus guidelines?**   Very familiar | 41 (56.2) | 67 (54.9) | 0.535 |
| 1. **Number of EoE-related educational activities attended in the previous 3 years**   <3  ≥3 | 40 (54.8)  33 (45.2) | 58 (47.5)  64 (52.5) | 0.327 |
| 1. **New EoE patients do you diagnose annually**   **<6**  **≥6** | 50 (68.5)  23 (31.5) | 74 (60.7)  48 (39.3) | 0.271 |
