# Supplementary material for: Adherence to clinical guidelines for the evaluation and management of eosinophilic esophagitis among gastroenterologists in the Arab countries
Source: Front Pediatr. 2025 Apr 10;13:1521266. doi: 10.3389/fped.2025.1521266 (PMC12018459; doi:10.3389/fped.2025.1521266)
Supplement: Supplementary file 2 [file Table2.docx]

**Supplementary table 2.** EoE practice patterns based on practice setting

| Guidelines recommendations | University-based  n = 56 | Non- University-based  n = 134 | P-value |
| --- | --- | --- | --- |
| 1. No need for PPI trial prior to diagnosis of EoE? (%) | 39 (69.6) | 98 (70.5) | 0.905 |
| 1. Number 4 of esophageal biopsies to diagnose of EoE (%) | 43 (76.8) | 82 (59.0) | **0.019** |
| 1. Biopsies from Proximal and distal esophagus (%) | 34 (60.7) | 72 (51.8) | 0.258 |
| 1. Place biopsies from different locations in different jars (%) | 46 (82.1) | 113 (81.3) | 0.890 |
| 1. Biopsies from stomach and duodenum on initial exam (%) | 46 (82.1) | 113 (81.3) | 0.890 |
| 1. Use of cut point of ≥15 eosinophils /hpf for diagnosis (%) | 44 (78.6) | 109 (78.4) | 0.981 |
| 1. Necessity for symptoms + positive biopsy + exclusion of secondary causes | 7 (12.5) | 24 (17.3) | 0.410 |
| 1. PPI monotherapy as first line treatment (%) | 33 (58.9) | 89 (64.0) | 0.506 |
| 1. Involvement of patients (or parents) in the decision-shared process (%) | 48 (85.7) | 118 (84.9) | 0.884 |
| 1. Assess both symptoms and histology as markers of treatment response (%) | 36 (64.3) | 80 (57.6) | 0.386 |
| 1. Use of maintenance therapy after steroid response (%) | 43 (76.8) | 97 (69.8) | 0.326 |
| 1. Dilation of severe esophageal strictures seen during first endoscopy (%) | 17 (30.4) | 36 (25.9) | 0.527 |
