# Supplementary material for: Adherence to clinical guidelines for the evaluation and management of eosinophilic esophagitis among gastroenterologists in the Arab countries
Source: Front Pediatr. 2025 Apr 10;13:1521266. doi: 10.3389/fped.2025.1521266 (PMC12018459; doi:10.3389/fped.2025.1521266)
Supplement: Supplementary file 3 [file Table3.docx]

**Supplementary table 3.** EoE practice patterns based on number of EoE activities attended

| Guidelines recommendations | < 3 activities  n = 94 | ≥ 3 activities  n = 96 | P-value |
| --- | --- | --- | --- |
| 1. No need for PPI trial prior to diagnosis of EoE? (%) | 66 (67.3) | 71 (73.2) | 0.372 |
| 1. Number 4 of esophageal biopsies to diagnose of EoE (%) | 58 (59.2) | 69 (69.1) | 0.150 |
| 1. Biopsies from Proximal and distal esophagus (%) | 51 (52.0) | 55 (56.7) | 0.514 |
| 1. Place biopsies from different locations in different jars (%) | 78 (79.6) | 81 (83.5) | 0.481 |
| 1. Biopsies from stomach and duodenum on initial exam (%) | 78 (79.6) | 81 (83.5) | 0.481 |
| 1. Use of cut point of ≥15 eosinophils /hpf for diagnosis (%) | 73 (74.5) | 80 (82.5) | 0.175 |
| 1. Necessity for symptoms + positive biopsy + exclusion of secondary causes | 11 (11.2) | 20 (20.6) | 0.073 |
| 1. PPI monotherapy as first line treatment (%) | 57 (58.2) | 65 (67.0) | 0.202 |
| 1. Involvement of patients (or parents) in the decision-shared process (%) | 84 (85.7) | 82 (84.5) | 0.817 |
| 1. Assess both symptoms and histology as markers of treatment response (%) | 57 (58.2) | 59 (69.8) | 0.705 |
| 1. Use of maintenance therapy after steroid response (%) | 64 (65.3) | 76 (78.4) | **0.043** |
| 1. Dilation of severe esophageal strictures seen during first endoscopy (%) | 19 (19.4) | 34 (35.1) | **0.014** |
