# Supplementary material for: Adherence to clinical guidelines for the evaluation and management of eosinophilic esophagitis among gastroenterologists in the Arab countries
Source: Front Pediatr. 2025 Apr 10;13:1521266. doi: 10.3389/fped.2025.1521266 (PMC12018459; doi:10.3389/fped.2025.1521266)
Supplement: Supplementary file 4 [file Table4.docx]

**Supplementary table 4.** EoE practice patterns based on duration of practice

| Guidelines recommendations | Junior consultants  n =98 | Senior consultants  n = 92 | P-value |
| --- | --- | --- | --- |
| 1. No need for PPI trial prior to diagnosis of EoE? (%) | 73 (74.5) | 63 (98.5) | 0.359 |
| 1. Number 4 of esophageal biopsies to diagnose of EoE (%) | 62 (63.3) | 60 (65.2) | 0.779 |
| 1. Biopsies from Proximal and distal esophagus (%) | 52 (53.1) | 51 (55.4) | 0.743 |
| 1. Place biopsies from different locations in different jars (%) | 84 (85.7) | 70 (76.1) | 0.091 |
| 1. Biopsies from stomach and duodenum on initial exam (%) | 81 (82.7) | 74 (80.4) | 0.693 |
| 1. Use of cut point of ≥15 eosinophils /hpf for diagnosis (%) | 87 (88.8) | 63 (68.5) | **0.001** |
| 1. Necessity for symptoms + positive biopsy + exclusion of secondary causes | 16 (16.3) | 15 (16.3) | 0.997 |
| 1. PPI monotherapy as first line treatment (%) | 66 (67.3) | 54 (58.7) | 0.217 |
| 1. Involvement of patients (or parents) in the decision-shared process (%) | 79 (80.6) | 82 (89.1) | 0.103 |
| 1. Assess both symptoms and histology as markers of treatment response (%) | 61 (62.2) | 52 (56.6) | 0.422 |
| 1. Use of maintenance therapy after steroid response (%) | 67 (68.4) | 69 (75) | 0.311 |
| 1. Dilation of severe esophageal strictures seen during first endoscopy (%) | 27 (27.6) | 24 (26.1) | 0.820 |
