# Supplementary material for: Adherence to clinical guidelines for the evaluation and management of eosinophilic esophagitis among gastroenterologists in the Arab countries
Source: Front Pediatr. 2025 Apr 10;13:1521266. doi: 10.3389/fped.2025.1521266 (PMC12018459; doi:10.3389/fped.2025.1521266)
Supplement: Supplementary file 5 [file Table5.docx]

| **Variables**  **Table 1. Overall characteristics of the respondents** | **Respondents = 190** |
| --- | --- |
| 1. **Practice setting** | |
| 1. Governmental, Tertiary care (University-based) | 56 (29.5%) |
| 1. Governmental, Tertiary care (Non-University based) | 72 (38%) |
| 1. Governmental, Secondary care | 29 (15%) |
| 1. Private practice | 33 (17.5%) |
| 1. **How many years in practice?** | |
| 1. 0-5 years 2. 6-10 years 3. 11-20 years 4. > 20 years | 65 (34%)  33 (17.5%)  48 (25%)  44 (23%) |
| 1. **Region of practice** | |
| Saudi Arabia | 122 (65%) |
| United Arab Emirates | 40 (21%) |
| Jordan | 4 (2%) |
| Syria | 4 (2%) |
| Algeria | 2 (1%) |
| Tunisia | 1 (0.5%) |
| Egypt | 1 (0.5) |
| Lebanon | 2 (1%) |
| Morocco | 2 (1%) |
| Oman | 5 (2.5%) |
| Kuwait | 5 (2.5%) |
| Sudan | 1 (0.5%) |
| Iraq | 1 (0.5%) |
| 1. **Your EoE practice population** | |
| Adults only | 55 (29%) |
| Children only (age ≤ 14 years) | 118 (62%) |
| Mixture of adults and children | 17 (9%) |
| 1. **Are you familiar’ with EoE consensus guidelines?** | |
| Very familiar | 104 (54.7%) |
| Somewhat familiar | 81 (42.6%) |
| Not familiar | 5 (2.7%) |
| 1. **Number of EoE-related educational activities attended in the previous 3 years** | |
| None | 23 (12%) |
| 1-2 | 73 (38.3%) |
| 3-4 | 51 (26.7%) |
| ≥ 5 | 43 (22%) |
| 1. **Areas of sub-specialization** | |
| General gastroenterology | 91 (48%) |
| Hepatology / Transplant Hepatology | 37 (19.5%) |
| Advanced Endoscopy | 41 (21.5%) |
| Inflammatory bowel disease | 55 (29%) |
| Motility disorders | 18 (9.5%) |
| Nutrition | 23 (12%) |
| 1. **New EoE patients do you diagnose annually** | |
| None | 5 (2.6%) |
| 1 - 5 | 116 (61%) |
| 6 - 15 | 56 (29.5%) |
| 16 - 25 | 10 (5.4%) |
| > 25 | 3 (1.5%) |
