# Supplementary material for: Adherence to clinical guidelines for the evaluation and management of eosinophilic esophagitis among gastroenterologists in the Arab countries
Source: Front Pediatr. 2025 Apr 10;13:1521266. doi: 10.3389/fped.2025.1521266 (PMC12018459; doi:10.3389/fped.2025.1521266)
Supplement: Supplementary file 6 [file Table6.docx]

**Table 2. Diagnosis of eosinophilic esophagitis (Questions 9 - 17)**

| **Variables** | **Respondents = 190** | | |
| --- | --- | --- | --- |
| 1. **Symptoms would make you consider the diagnosis of EoE** | | | |
| 1. Heartburn | 59 (31%) | | |
| 1. Regurgitation | 43 (22.5%) | | |
| 1. Refractory reflux | 94 (49.5%) | | |
| 1. Chest pain | 88 (46%) | | |
| 1. Abdominal pain | 36 (19%) | | |
| 1. Dysphagia | 181 (95%) | | |
| 1. Odynophagia | 69 (36.4%) | | |
| 1. Food impaction | 166 (87.3%) | | |
| 1. Nausea | 27 (14%) | | |
| 1. Vomiting | 88 (46%) | | |
| 1. Weight loss / Failure to thrive | 80 (42%) | | |
| 1. Anemia | 26 (13.7%) | | |
| 1. Hematemesis | 28 (14.7%) | | |
| 1. Personal or family history of atopic disorders / food allergies | 127 (67%) | | |
| 1. **Endoscopic findings do you consider consistent with the diagnosis of EoE** | | | |
| 1. Esophageal rings | 167 (88%) | | |
| 1. Esophageal stricture | 147 (77.4%) | | |
| 1. Esophageal ulcer | 38 (20%) | | |
| 1. Esophageal nodule | 32 (17%) | | |
| 1. Esophageal mass | 9 (4.7%) | | |
| 1. Narrow caliber esophagus | 119 (62.5%) | | |
| 1. Linear furrows | 178 (94%) | | |
| 1. White plaques/exudates | 155 (81.5%) | | |
| 1. Erosive esophagitis | 51 (27%) | | |
| 1. Decreased mucosal vascularity | 61 (32%) | | |
| 1. Congested esophageal mucosa | 58 (30.5%) | | |
| 1. Mucosal tears after passing the endoscope | 76 (40%) | | |
| 1. Hiatal hernia | 8 (4%) | | |
| 1. Normal appearing esophagus | 52 (27%) | | |
| 1. **Do you require that a patient is on PPI diagnosis of EoE?** | | | |
| 1. Yes | 58 (20.5%) | | |
| 1. No | 137 (72%) | | |
| 1. **Number of esophageal biopsies to diagnose of EoE** | | | |
| 1. 2 | 7 (3.6%) | | |
| 1. 4 | 63 (33%) | | |
| 1. 6 | 92 (48.4%) | | |
| 1. 8 | 24 (12.6%) | | |
| 1. > 8 | 9 (4.7%) | | |
| 1. **From where in the esophagus do you take biopsies?** | | | |
| 1. Proximal esophagus | 136 (71.5%) | | |
| 1. Mid esophagus | 158 (83%) | | |
| 1. Distal esophagus | 175 (92%) | | |
| 1. **Do you put biopsies in different pathology jars?** | | | |
| 1. Yes | 159 (83.5%) | | |
| 1. No | 36 (19%) | | |
| 1. **Do you get biopsies from stomach and duodenum?** | | | |
| 1. Stomach only | 31 (16.3%) | | |
| 1. Duodenum only | 5 (2.6%) | | |
| 1. Both stomach and duodenum | 159 (83.5%) | | |
| 1. **Cut off eosinophils/high-power field do you use for diagnosis of EoE** | | | |
| 1. 10 | 6 (3%) | | |
| 1. 15 | 149 (78.4%) | | |
| 1. 20 | 21 (10.5%) | | |
| 1. 25 | 10 (5.2%) | | |
| 1. I don’t use a specific cut off point | 9 (4.67% | | |
| 1. **Which of the following are necessary to diagnose EoE?** | Not necessary | Helpful, but not necessary | Necessary |
| 1. Clinical symptoms of esophageal dysfunction | 10 (5.2%) | 129 (68%) | 51 (26.8%) |
| 1. Allergy testing | 67 (35.3%) | 119 (62.7%) | 4 (2%) |
| 1. Barium swallow study | 80 (42.1%) | 105 (55.3%) | 5 (2.6%) |
| 1. Eosinophil-predominant inflammation on esophageal biopsy | 2 (1%) | 17 (9%) | 173 (90%) |
| 1. Peripheral eosinophilia | 75 (39.5%) | 110 (58%) | 5 (2.5%) |
| 1. Exclusion of secondary causes of esophageal eosinophilia | 21 (11%) | 70 (37%) | 99 (52%) |
| 1. Ruling out gastroesophageal reflux disease with pH testing | 93 (49%) | 83 (43.6%) | 14 (7.4%) |
| 1. No clinical response to a PPI trial | 52 (27.5%) | 111 (59.5%) | 23 (12%) |
| 1. Personal or family history of atopic disorders / food allergies | 13 (6.4%) | 173 (91.6%) | 4 (2%) |
